# Sex and gender differences in caregiving burden experienced by family caregivers of persons with dementia: A systematic review

**DOI:** 10.1371/journal.pone.0231848

**Published:** 2020-04-20

**Authors:** Chen Xiong, Melissa Biscardi, Arlene Astell, Emily Nalder, Jill I. Cameron, Alex Mihailidis, Angela Colantonio

**Affiliations:** 1 Rehabilitation Sciences Institute, Faculty of Medicine, University of Toronto, Toronto, Canada; 2 Toronto Rehabilitation Institute-University Health Network, Toronto, Canada; 3 Aquired Brain Injury Research Lab, University of Toronto, Toronto, Canada; 4 Department of Occupational Science & Occupational Therapy, University of Toronto, Toronto, Canada; 5 Dalla Lana School of Public Health, University of Toronto, Toronto, Canada; University College London, UNITED KINGDOM

## Abstract

**Objectives:**

Much is known about the demands of caregiving for persons with dementia (PWD) and its effects on family caregivers, however sex and gender aspects have received less attention. We synthesized the evidence on sex and gender distinctions in: (1) the caregiving burden and (2) the impact of caregiving on the physical and mental health of family caregivers of PWD.

**Design:**

Systematic review.

**Data sources:**

Medline, Embase, PsycINFO and Cumulative Index to Nursing and Allied Health Literature between January 2007 and October 2019 were searched.

**Eligibility criteria for selecting studies:**

Included studies met the following criteria: (1) examine experiences and/or impacts of caregiving among family caregivers of individuals with any form of dementia; (2) report sex and/or gender distribution of study population and/or report results stratified by sex and/or gender, and (3) include both male and female family caregivers.

**Data extraction and synthesis:**

Two independent reviewers extracted the data and assessed risk of bias using the Critical Appraisal Skills Program checklist and National Institutes of Health Quality Assessment Tool for Observational Cohort and Cross-sectional Studies. Data were synthesized using a narrative synthesis approach.

**Results:**

A total of 22 studies were included. Caregiving burden was measured using various methods. A majority of studies reported higher burden among females. All studies that did not report a sex and gender difference in caregiving burden accounted for confounders. Findings on sex and gender differences on physical and mental health conditions were inconsistent with most studies failing to account for confounders in their analyses.

**Conclusions:**

Current evidence on sex and gender differences in caregiving burden, mental and physical health is limited. Findings suggest presence of sex and gender differences in caregiving burden. Given the variety of mental and physical health constructs that were examined, further research is required to substantiate the evidence.

*PROPSERO Registration Number*: *CRD 42018070032*.

## Introduction

Dementia, which refers to a number of conditions that produce acquired cognitive decline [1], is a major global public health concern. More than 47 million individuals are living with dementia related conditions worldwide and that number is expected to increase to more than 131 million by 2050 [2]. In Canada, the direct and indirect health care system and caregiver costs associated with dementia currently exceed $10 billion [3] per annum. In addition to cognitive decline, persons with dementia (PWD) may also experience behavioral and psychological disturbances such as depressive mood, anxiety, restlessness, and agitation [4, 5].

With the rising prevalence of dementia, an increasing number of aging family members are providing care for PWD [6, 7]. In 2011, Canadian family caregivers provided more than 19 million unpaid hours of care, a number that is projected to double by 2031 [3]. Despite the benefits of home care such as the presence of kinship and delay of unfavorable health outcomes, caregiving remains a stressful experience and places a significant burden on family caregivers [8–11]. Conceptualized as a multidimensional response to the physical, psychological, emotional, social and financial stressors associated with the caregiving experience, caregiver burden had been hypothesized as an acute reaction to the introduction of new care demands and intensification of existing ones [12]. To date most research on measures of caregiving burden has been quantitative, providing tools that are easily adapted within clinical settings [13] and valuable information for evidence-based intervention programs. However, these measures may fail to capture the breadth of elements that comprise the multi-faceted concept of burden [13]. As such, this review will also include qualitative examinations of caregiving burden with the goal of achieving a more comprehensive understanding of caregiving burden in the context of family caregiving and dementia.

With the progressive decline experienced by PWD, family caregivers who face difficulties adapting or modifying their care strategies experience a significant level of caregiver burden [14]. Previous research has shown that attributes of both caregiver and care recipient play a role in mediating caregiving outcomes [15]. In particular, older age, lower socioeconomic status and lower education level have all been associated with higher levels of caregiver burden [16]. Additionally, care recipient attributes including dementia severity, presence of behavioral disturbances, extent of personality change as well as presence of psychiatric symptoms are also identified as factors that contribute to an increased level of caregiving burden [16].

Caregiving burden can have devastating and long term effects on the physical, social emotional as well as financial status of family caregivers of PWD [17, 18]. Previous work has shown an association between caregiving burden and psychological distress, including depression, as well as physical conditions such as hyperlipidemia and hyperglycemia [19–21]. More specifically, caregivers of PWD demonstrate a high prevalence of self-reported depression and reduced physical health including disrupted sleep patterns, lowered immunity and early transition to frailty syndrome [19, 22].

While much is known about caregiving burden and its effects on family caregivers and their care recipients, there has been little exploration of possible sex and gender differences between male and female family caregivers of PWD. At present, females are the predominant providers of informal care for family members with chronic medical conditions including dementia [23]. Within the context of this review, sex represents a set of biological attributes in humans associated with physical and physiological features, while gender constitutes the socially constructed roles, behaviors, expressions and identities of girls, women, boys, men and gender diverse individuals [24]. Despite being distinct constructs, it is important to recognize that sex and gender intersect and are interrelated [25]. Hence, both constructs will be referred to as ‘sex and gender’ for the remainder of the review.

Previous analyses of sex and gender differences among caregivers have shown a considerable distinction with respect to physical and psychosocial health status [26]. Specifically, female caregivers report higher levels of depressive symptomatology and are at a higher risk for clinical depression compared to their male counterparts [27]. Additionally, female caregivers are found to report poorer physical health and more emotional distress due to caregiving compared to their male counterparts [15, 28, 29]. While prior reviews in the field of caregiving burden provided pioneering perspectives on potential sex and gender differences among caregivers of PWD [16, 30], there has not been an evidence synthesis dedicated towards uncovering the sex and gender differences within this population.

To address this research gap, the objectives of this systematic review were to: (1) examine any sex and gender distinctions in the nature and level of caregiving burden experienced by family caregivers of persons with dementia, and (2) determine the sex and gender differences in the impact of caregiving on specific physical and mental health constructs among family caregivers of PWD.

## Methods

The systematic review was conducted based on a previously peer-reviewed protocol registered with the International Prospective Register of Systematic Reviews (PROSPERO) (registration number CRD 42018070032) and published in an open access journal [31]. The presentation of the findings was guided by the Preferred Reporting Items for Systematic Reviews and Meta-Analyses (PRISMA) checklist [32].

### Search strategy

Due to the extensive number of studies identified within the searched databases and the limited empirical evidence regarding the impact of search and including earlier works on systematic review findings [33], our search strategy covered a publication period from January 2007 and October 2019 within the following databases:

1. MEDLINE (including Medline in Process and other non-indexed citations, ePubs and Medline Daily);
2. Cumulative Index to Nursing and Allied Health Literature (CINAHL);
3. Embase;
4. PsycINFO.

Please refer to the published protocol for details on the data searches and MeSH terms used for each database [31].

### Inclusion and exclusion criteria

Given the importance of disaggregating the data by sex and gender when conducting a sex and gender analysis [34], studies included in the review met the following criteria: (1) examine the experiences and/or impacts of caregiving among family caregivers of individuals with any form of dementia; (2) report sex and/or gender distribution of study population and/or report and discuss results stratified by sex and/or gender, and (3) include both male and female family caregivers of persons with dementia. Studies that (1) include both family and formal caregivers but do not stratify findings by caregiver type, (2) do not report results specifically for care recipients with some form of dementia or (3) examine the effects of various interventions on caregiving burden were excluded. Additionally, the following study designs/formats were excluded: case reports or public reports, theses, abstracts, conference materials, editorials and commentaries.

### Data extraction: Selection and coding

Two researchers (CX and MB) independently screened study titles and/or abstracts as well as reviewed full texts of manuscripts to determine fulfillment of the inclusion criteria. Discrepancies in opinion were resolved through discussion with a third researcher (AC). A standardized form was used to assess study quality and synthesize study findings from the included studies. Extracted information included the following: (1) author and publication year, (2) study setting and design, (3) study location, (4) information of the study population and demographic characteristics, (5) study results relating to caregiving experiences (i.e. caregiving burden and impacts on physical and/or mental health), (6) details on the methodology used to gather these experiences, (7) the statistical approach used and confounders, (9) information on sex and gender differences and (10) information on the risk of bias assessment. Two reviewers (CX and MB) extracted the data independently and a third reviewer (AC) reviewed the quality of data extraction and mediated a resolution in cases of disagreement through follow-up discussions with the reviewers.

### Risk of bias (Quality) assessment

Quality assessment of the studies was conducted independently by two reviewers (CX and MB). Qualitative studies were assessed using the Critical Appraisal Skills Program (CASP) qualitative checklist and consisted of the following steps: (i) assessment of potential sources of bias through a series of 10 questions related to the results, their validity and impact, and (ii) responding to each question as “Yes”, “Cannot Tell” and “No” [35]. Quantitative studies were assessed using the National Institutes of Health (NIH) Quality Assessment Tool for Observational Cohort and Cross-sectional Studies through the following process: (i) assessment of potential sources of bias through a series of 10 criteria applicable to the study, and (ii) grading the presence of potential biases as “Yes” “Cannot Determine”, “No”, “Not Reported” or “Not Applicable” [36]. Following the grading of each study, the overall level of potential bias was summarized: “++” when all or most of the quality criteria were fulfilled and the study classified as “high quality”; “+” when some of the criteria were fulfilled and the study classified as “moderate quality”; “−” when few or no criteria were fulfilled and the study classified as “low quality”. Studies that were classified as “low quality” were excluded from the review.

### Data synthesis

The included studies were analyzed using a narrative synthesis approach following the Guidance for Narrative Synthesis in Systematic Reviews [37]. Specifically, textual descriptions, tabulation as well as grouping and clustering were employed in the analyses. Synthesis of the extracted data involved the summarization and explanation of the sex and gender differences for the included studies. In addition, the quality of the included studies was described as part of the narrative synthesis. While a plan was in place to investigate the pooled effect of sex and gender on various aspects of caregiving experiences, the high heterogeneity between the included studies, concerning methodology (statistical methods, type and method of assessment of caregiving experiences), population (age, sex and gender, dementia type, etc.) as well as study settings (country, recruitment locations, etc.) ruled out meta-analysis.

### Patient and public involvement

Patients and the public were not involved in this review.

## Results

**Fig 1:**
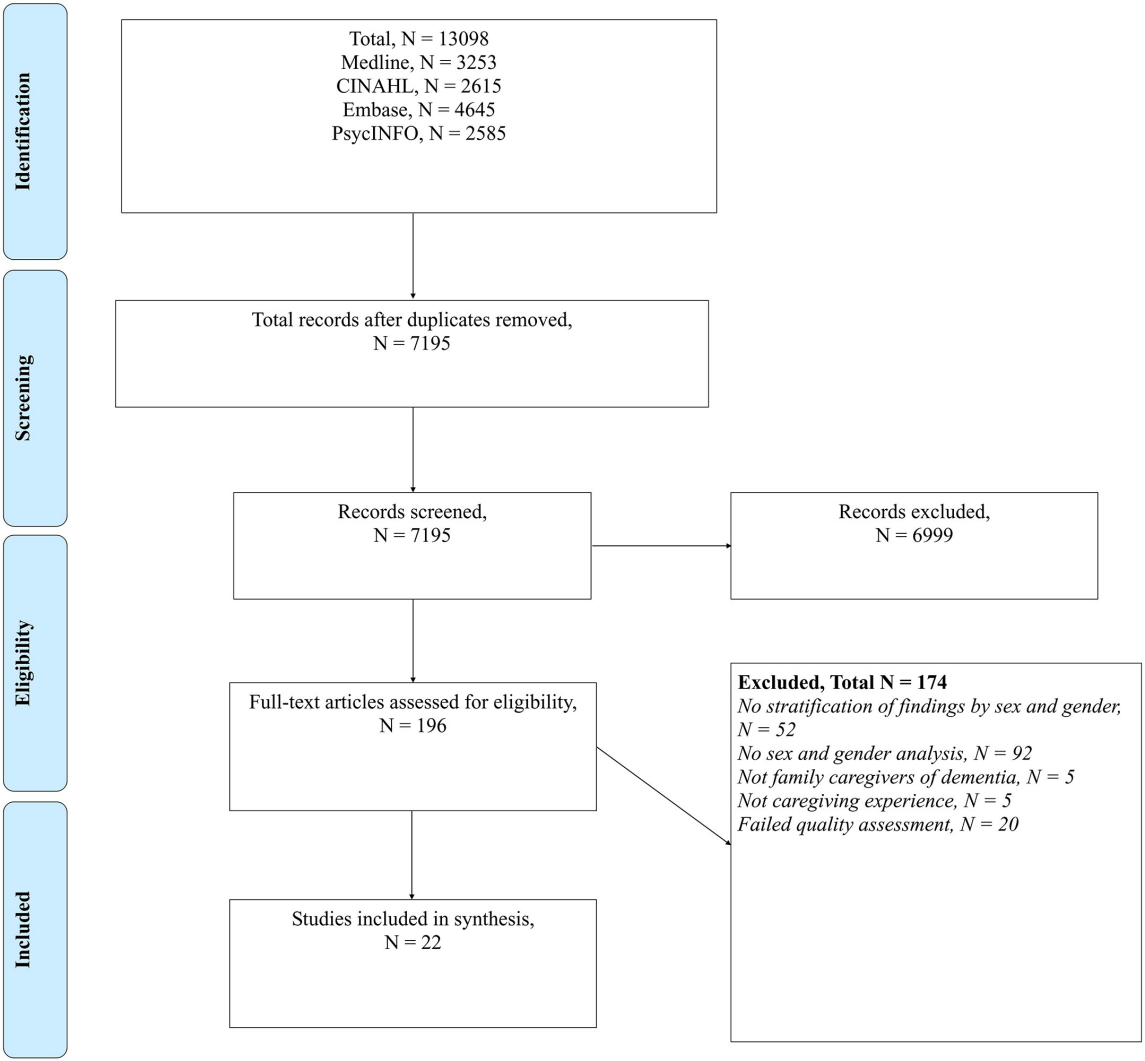
PRISMA diagram.

The searches yielded a total of 13098 records, from which 7195 records remained after the duplicates were removed. Of the 7195 records, 196 met the criteria for a full-text screen. As part of the full-text screen, articles were excluded if they did not stratify findings by sex and/or gender, did not conduct a sex and gender analysis, did not involve family caregivers of dementia or did not examine caregiving experience. Of the remaining 42 studies that were included for the quality assessment, 20 of the studies were of ‘low’ quality and were excluded. A total of 22 studies, all of ‘moderate’ quality except for one which was classified as ‘high’ quality, were included (Fig 1) [38–59]. These were divided into 18 quantitative studies and four qualitative studies which are reported in two sections below.

### Quantitative studies

#### Study characteristics

A summary of the 18 included quantitative studies is presented in Table 1. Of the quantitative studies, all were of a cross-sectional design. With respect to study setting, 15 studies were community-based [38–45, 47–50, 56–58], two were based in the clinic [46, 51] and one was based in both the community and clinic [52]. The type of dementia experienced by the care recipients varied among studies. Seven of the studies included only persons with Alzheimer’s disease; another seven studies included persons with Alzheimer’s disease and other types of dementia such as vascular dementia, Lewy-body disease, frontotemporal dementia among others. The remaining four studies did not report information on the type of dementia.

**Table 1:** Findings of all included quantitative studies.

#	Author Date Country Design Sample by Inclusion criteria (IC) Exclusion criteria (EC)	Population Sample size Age (mean±(SD)/ range), yrs Sex/Gender (%M) Dementia Type Assessment criteria Frequencies (%)	Outcome definition Sources	Analyses Methodology	Results Unadjusted Adjusted	Adjustment (Confounders) Notes Limitations
1	Akpinar et al. 2011 Turkey Cross-sectional Community IC: family member of PWD; primary caregiver that scored <26 on MMSE EC: CR diagnosed with other dementias	N = 192 Age: M: 74.26±8.27 F: 75.82±8.91 Sex/gender: 37.5% M AD NR	**Burden** CBI	*t*-tests	**Unadj. *t*-tests** Significantly *higher levels of overall* (p = 0.002), *time-dependence* (p = 0.04), *developmental* (p = 0.002), *physical* (p = 0.01) and *social* (p = 0.045) burden among females Emotional burden NS	NR Limitations: did not take into account possible confounders in analyses
2	Chappell et al. 2016 Canada Cross-sectional Community IC: spoke English, family member, at least 3 hours of care per week, care recipient taking ChEI and living at home EC: NR	N = 873 Age: 67.03 (range: 28–93) Sex/gender: 31.3% M AD, VaD and other Physician diagnosis AD: 59.2% VaD: 12.9% Others: 27.9%	**Burden** ZBI **Self-esteem** Rosenberg Scale of Self-Esteem	*t*-tests	**Unadj. *t*-tests** Significantly *higher burden* (p<0.01) among females Self-esteem NS	NA Limitations: did not account for confounders, only included care recipients taking ChEI
3	Conde-Sala et al. 2010 Spain Cross-sectional Community IC: informed consent of CR and CG, CR with clinical diagnosis of AD and MMSE between 10–28 EC: NR	N = 251 Age: Spouses: 75.38±7.35 Adult children: 79.56±5.75 Sex/gender: 34% M AD or probable AD DSM-IV and NINCDS-ADRDA criteria Minimal: 48.2% Mild: 38.6% Moderate: 10.8%	**Burden** CBI	Mann-Whitney tests	**Unadj. Mann-Whitney-tests** Significantly *higher CBI scores* (p = 0.039) among wives	NA Limitations: did not account for confounders
4	Davis et al. 2012 USA Cross-sectional Community IC: CR to be community dwelling, in committed relationship and have partner willing to provide information at baseline EC: NR	N = 162 Age: 73.28±9 Sex/gender: 59.9% M Probable/possible AD MMSE and Blessed Dementia Scale Probable AD: 76.5% Possible AD: 12.4% Mixed AD: 11.1%	**Burden** CEQ **Intimacy experience** EOIPS	*t*-tests Linear regression	**Unadj. *t*-tests** Significantly *higher CEQ scores* (p = 0.0002) among females EOIPS items NS **Adj. multivariate analyses** **(β, Standard error)** CEQ (ref: M): 1.774, 0.552; p<0.01 CEQ (post-hoc) (ref: M): 2.145, 0.693; p<0.01	CG satisfaction with intimacy, AD severity Limitations: did not account for other potential confounders
5	Ducharme et al. 2011 Canada Cross-sectional Community IC: main person responsible for relative >65 years of age with AD in past 9 months EC: receiving psychotherapy or in support group	N = 122 Age: 61.42±13.62 Sex/gender: 20.5% M AD Formulated by geriatricians and neurologists NR	**Psychological distress** Psychological distress index **Family conflicts** Family caregiver conflict scale **Self-efficacy** Revised Scale for caregiving self-efficacy	ANOVA	**Unadj. ANOVA** Significantly *more family conflicts* (p≤0.01) and *higher psychological distress* (p≤0.01) among females Significantly *lower scores on controlling disturbing thoughts* (p≤0.01) among females	NA Limitations: did not account for confounders
6	Lee et al. 2019 USA Cross-sectional Community IC: NR EC: CR bedbound and has MMSE = 0 or has no diagnosis of dementia and MMSE >23	N = 632 Age: 60.5±13.36 Sex/gender: 22% M NR	**Depressive symptoms** CES-D	Chi-square tests Logistic regression	**Unadj. chi-square tests** Females reported significantly higher levels of *burden* compared to males (p = 0.007) **Adj. multivariate analyses** **OR (95% CI); p-value** Depressive symptoms (CES-D≥10, ref: M): 2.02 (1.2–3.38); <0.001	CR cognitive function & problem behavior, CG age, ethnicity, education, financial difficulty, employment status, marital status, self-rated health, relationship to CR, length of caregiving, social support, leisure engagement satisfaction Limitations: secondary data analyses
7	Losada et al. 2010 Spain Cross-sectional Community IC: primary source of help, >1 caregiving hour per day for >3 months EC: NR	N = 288 Age: 59.63±12.6 Sex/gender: 20.8% M AD and other dementias NR AD: 58.4% Others: 41.6%	**Guilt** Caregiver Guilt Questionnaire	*t*-tests	**Unadj. *t*-tests** Significantly higher scores on factors *‘guilt about neglecting other relatives’* (p<0.01), ‘*guilt about having negative thoughts toward others*’ (p<0.05) and *total scores* (p<0.05) among females	NA Limitations: did not account for confounders; cultural impact on guilt not examined
8	Mills et al. 2009 USA Cross-sectional Community IC: ≥55 years of age, spouse living at home with dementia spouse, not take anticoagulant medication EC: NR	N = 81 Age: 71.7 Sex/gender: 20.8% M NR CDR scale High CDR: 49.4% Low CDR: 50.6%	**Role overload stress** Pearlin Role Overload scale **Sleep** WASO, sleep efficiency, AHI, slow wave sleep **Coagulation and Inflammation** D-dimer, IL-6	ANCOVA MANCOVA	**Adj. ANCOVA** Significantly higher *role overload stress* (p<0.01) among females compared to males Significantly higher *D-dimer* and *IL-6* levels in males caring for spouses with high CDR **Adj. MANCOVA** Significantly higher *WASO*, worse *AHI* and lower *slow wave sleep* in males caring for spouses with high CDR Sleep efficiency NS	Age, BMI, dementia severity Limitations: only one blood sample taken for diurnal markers (e.g. IL-6)
9	Papastavrou et al. 2009 Cyprus Cross-sectional Community IC: frequent contact with CR, care for ≥ 1 year, absence of psychiatric illness/mental disability EC: NR	N = 172 Age: NR Sex/gender: 23.3% M AD NR	**Burden** ZBI **Depression** CES-D	*t*-tests	**Unadj. *t*-tests** Significantly higher *ZBI* (p = 0.048) and *CES-D* (p = 0.011) scores among women Significant higher scores in ZBI items of *relational deprivation* (p = 0.002) among women Other factors NS For CRs living at home *ZBI* and *CES-D* NS Significantly higher scores in ZBI items of *relational deprivation* (p = 0.035) and lower scores in ZBI items of *management of care* (p = 0.003) among females	NA Limitations: did not consider confounders
10	Pattanayak et al. 2010 India Cross-sectional Clinical IC: CR ≥60 years old with AD, at least 1 year of illness, CG ≥18 years old, providing care for ≥1 year, willing to participate EC: presence of major illness in CR, CG or other family	N = 32 Age: 53.94±16.16 Sex/gender: 43.75% M AD DSM-IV NR	**Burden** Burden Assessment Schedule	*t*-tests Multiple regression	**Unadj. *t*-tests** Significantly higher mean scores in *total burden* (p = 0.04), *physical and mental health* (p = 0.01), *spouse-related* (p = 0.00) and *caregiver’s routine* (p = 0.01) among females Other factors NS **Adj. multivariate analyses** Burden: NS	Education, relation to CR, CR gender, Hindu Mental State Examination score Limitations: small sample
11	Posyti et al. 2012 Finland Cross-sectional Community IC: NR EC: NR	N = 335 Age: 77±6.2 (M), 78.4±5.6 (F) Sex/gender: 38.2% M NR	**Burden** ZBI **Depression** Geriatric Depression Scale **Comorbidity** CCI	Mann-Whitney tests Logistic regression	**Unadj. Mann-Whitney tests** Significantly higher *burden* (p<0.001) and *points in depression scale* (p = 0.0025) among females. Significantly more *comorbidity* (p<0.001) among males. **Adj. multivariate analyses** **OR (95% CI); p-value** High burden (ZBI>40 points, ref: F): 0.33 (0.18–0.62); p<0.001	CR and CG age, CCI, CG education and home care services use, CR MMSE, NPI and Cornell scale points Limitations: no indication of IC/EC and dementia type
12	Prince et al. 2012 Various countries Cross-sectional Community IC: NR EC: NR	N = 673 Age: NR Sex/gender: 33% M NR	**Burden** ZBI	*t*-tests Regression modelling	**Unadj. *t*-tests** Significantly higher *burden* scores among women in Cuba and urban Peru. All other countries NS **Adj. multivariate analyses** **Pooled fixed effect adjusted mean difference (95% CI)** ZBI score (ref: M): -2.5 (-5.3–0.2)	CG age, marital status, relationship, psychological morbidity, CR age, gender, severity of behavioural/psychological symptoms, co-resident number, time spent assisting with ADLs Limitations: Lack of info on population, significance levels unspecified
13	Sutcliffe et al. 2016 United Kingdom Cross-sectional Community IC: CR ≥65 years old, definite/probable dementia diagnosis, <24 on Standardized MMSE, receive community service, have CG that lived with or visited ≥2 times monthly	N = 81 Age: 65.4±12.2 Sex/gender: 46% M NR Severe dementia: 30.3% Moderate dementia: 50% Mild dementia: 19.7%	**Burden** ZBI (high vs. low)	Chi-square tests Logistic regression	**Unadj. chi-square tests** Burden (high vs low): NS **Adj. multivariate analyses** **OR (95% CI); p-value** Burden (ref: M): 5.46 (1.37–21.79); p = 0.016	CR relationship, CR NPI severity, receipt of informal support, supervision of CR by CG Limitations: age not accounted for in analyses
14	Sutcliffe et al. 2017 8 European countries Cross-sectional Community IC: CR ≥65 years old, diagnosis of dementia, <24 on Standardized MMSE, receive community service, have CG that lived with or visited ≥2 times monthly	N = 1223 Age: 64.7±13.4 Sex/gender: 31.4% M AD, VaD, Mixed, others NR AD: 65% VaD: 19.6% Mixed: 7.1% Others: 8.3%	**Burden** ZBI (high vs. low)	Chi-square tests Logistic regression	**Unadj. chi-square tests** Females reported significantly higher levels of *burden* compared to males (p<0.001) **Adj. multivariate analyses** **OR (95% CI); p-value** Burden (ref: M): NS	CG relationship, living arrangements, CR age, gender, standardized MMSE, Katz ADL score, NPI severity, Cornell depression score, CCI, caregiving hours, informal support, country Limitations: missing data
15	Takai et al. 2011 Japan Cross-sectional Clinical IC: NR EC: NR	N = 118 Age: 60.9±14 Sex/gender: 40.7% M AD, VaD FTD, dementia with Lewy bodies, mixed Diagnostic criteria based on NINCDS-ARDA, NINDS-AIREN, Lund and Manchester Groups and consensus guidelines AD: 77.9% VaD: 11% FTD: 4.2% LBD: 2.5% Mixed: 4.2%	**Quality of life** World Health Organization Quality of Life 26 questionnaire **Burnout** Pines Burnout Measure **Depression** BDI, second edition	F-tests	**Unadj. F—tests** Significantly higher *BDI* (p = 0.02) and *burnout measure* scores (p = 0.01) among females Significantly higher *psychological quality of life* (p = 0.05) scores among males	NA Limitations: Did not account for potential confounders
16	Ulstein et al. 2017 Norway Cross-sectional Clinical and community IC: CR living at home, fulfilled ICD-10 criteria of dementia and had weekly face to face contact with CG EC: CG who took part in support programs	N = 196 Age: 63.8±13 Sex/gender: 35% M NR ICD-10 criteria NR	**Burden** Relative Stress Scale	*t*-tests Linear regression	**Unadj. *t*-tests** Burden: NS **Adj. multivariate analyses** **β; p-value** Overall burden (ref: F): NS Emotional distress subscale (ref: F): -0.13; p = 0.03 Social distress subscale (ref: F): NS Negative feelings subscale (ref: F): NS	NPI score, Disability Assessment for Dementia, Hours caring per week, relationship with CR, daily contact Limitations: Unsure if there are other variables included in model
17	Valimaki et al. 2009 Finland Cross-sectional Community CR IC: 65+ years, very mild or mild AD, informed consent CR EC: NR	N = 170 Age: 71.6±7.2 Sex/gender: 37.1% M Very mild to mild AD Clinical dementia rating Very mild: 0.5 Mild: 1 NR	**Depression** BDI **Distress** GHQ **Sense of coherence** NR **HRQoL** 15D questionnaire and VAS	*t*-tests Linear regression	**Unadj. *t*-tests** Significantly *higher BDI* (p≤0.001) and *GHQ* (p = 0.016) scores among females compared to males Significantly *lower SOC* (p<0.001) in females compared to males HRQoL NS **Adj. multivariate analyses (β, 95% CI)** HRQoL: NS Sense of coherence factor 1 (ref: M): -3.536, -6.125–-0.947; p = 0.008	HRQoL Total amount of medication, BDI, GHQ Sense of coherence factor 1 Years of education, BDI, GHQ, income Limitations: unclear if sex/gender included in other regression models within study
18	von Kanel et al. 2019 Switzerland Cross-sectional Community IC: CG ≥55, English speaking, provide ≥20 hours per week of in home care, mild depressive symptoms EC: current treatment of malignancy, severe chronic illness, hypertension, psychiatric illness, participation in behavioral CG intervention, treatment with steroids, anticoagulants or non-selective beta-blocking	N = 134 Age: 74.1±8.3 Sex/gender: 21.6% M NR	**Self-rated Health** 12-item Short-Form Health Survey	Multinomial logistic regression	**Adj. multivariate analyses** **OR (95% CI); p-value** Self-rated health: NS	CG age, education, BMI, physical activity, alcohol consumption, smoking status, health problems, physical function, negative & positive affect, social support, CG stress Limitations: caregivers were mildly depressed; potential bias in race/ethnicity/education level

Abbreviations: AD—Alzheimer’s Disease; AHI—Apnea–Hypopnea Index; BDI—Beck Depression Inventory; CBI—Caregiver Burden Inventory; CCI—Charlson Comorbidity Index; CDR—Clinical Dementia Rating; CES-D—Centre of Epidemiologic Studies Depression Scale; CG—Caregiver; ChEI—Cholinesterase Inhibitor; CR—Care Recipient; EC—Exclusion Criteria; EOIPS: Experience of Intimacy with Partner Scales; FTD—Frontotemporal Dementia; GHQ—General Health Questionnaire; HRQoL—Health-related Quality of Life; IC—Inclusion Criteria; LBD—Lewy Body Dementia; MMSE—Mini Mental State Examination; NA—Not Applicable; NINCDS-ADRDA—National Institute of Neurological and Communicative Disorders and Stroke/Alzheimer’s Disease and Related Disorders Associations; NPI—Neuropsychiatric Inventory; NR—Not Reported; NS—Not significant; PDD—Parkinson’s Disease Dementia; PWD—Persons with Dementia; VAS—Visual Analogue Scale; WASO—Wake after Sleep Onset; ZBI—Zarit Burden Inventory

The 18 quantitative studies reported data from a total of 5735 (range 32–1223) caregivers. All of the studies reported information on sex/gender, and most studies reported the age of participants with the exception of two studies, which did not provide any information on the participants’ ages. The mean age in studies ranged from 53.9 [46] to 77.9 [47] years and the mean age among all reported samples was 66.7 years. The average percentage of men was 33.2% across all samples and the number of women exceeded that of men in all but one study [41]. With respect to caregiving relationships, 13 studies included a mix of children and spousal family caregivers [38–40, 42, 43, 45, 46, 48–52, 57] while five studies focused exclusively on spousal caregivers [41, 44, 47, 56, 58].

#### Caregiving burden

A range of methods were used to measure caregiving burden among family caregivers of PWD. Thirteen of the 18 included articles examined caregiving burden, with six using the Zarit Burden Scale (ZBI) [39, 45, 47–50] and two used the Caregiving Burden Inventory (CBI) [38, 40]. The other five studies used the Caregiving Experiences Questionnaire [41], Pearlin Role Overload Scale [44], Burden Assessment Schedule (BAS) [46], Pines Burnout Measure [51] and Relative Stress Scale (RSS) respectively [52]. With respect to confounding variables, eight studies incorporated adjustments for caregiver and care recipient demographic and clinical characteristics including relationship, education level, age, marital status, dementia severity, cognitive status and physical health [41, 44, 46, 47–50, 52]. A full list of confounders included in the studies can be found in Table 1. The remaining five studies did not adjust for any confounders in their analysis.

Overall, ten of the 13 studies directly assessing caregiver burden found higher reported burden or care-related distress among female caregivers [38–41, 44, 45, 47, 50, 51, 52]. These included three of the six studies that used the ZBI [39, 47, 50]. Additionally, one further study that utilized the ZBI found female caregivers scoring higher in items related to relational deprivation and lower in items relating to care management [45]. The other two studies that used ZBI did not find a significant difference between male and female caregivers [48, 49]. Studies that assessed caregiver burden using the CBI [38, 40], Pearlin Role Overload Scale [44], Caregiving Experiences Questionnaire [41], Pines Burnout Measure [51] and RSS [52] all found significantly higher scores for overall stress and/or burden among female caregivers while a single study that utilized the BAS did not identify any significant differences between the two sex and genders [46].

#### Mental health

Eight of the included studies examined the impact of caregiving on family caregivers’ mental health. Specifically, five studies investigated depression and all found significantly higher levels of depression in female caregivers compared to their male counterparts as measured by the Center for Epidemiologic Studies Depression Scale (CES-D) [45, 57], Beck Depression Inventory (BDI) [51, 56] and Geriatric Depression Scale (GDS) [47]. Additionally, female caregivers were found to have greater psychological stress [42, 56], more family conflicts [42], higher guilt [43], lower psychological quality of life [51], sense of coherence [56] and ability to control disturbing thoughts [42].

#### Physical health

With respect to family caregivers’ physical health, one study examined the impact of caregiving on sexual intimacy among spousal caregivers and found no significant difference in the impact of caregiving on sexual intimacy between male and female caregivers [41]. After adjusting for age, body mass index (BMI) and care recipient’s dementia severity, another study did find significant differences in sleep and inflammation biomarkers [44]. In particular, female caregivers experienced better sleep as measured by wake after sleep onset, Apnea Hypopnea Index, and slow wave sleep compared to male caregivers [44]. Specifically, female caregivers were found to experience less sleep apnea, more slow wave sleep and less time awake after sleep onset [44]. Male caregivers reported elevated levels of D-dimer and IL-6, which are biomarkers for increased thrombosis and inflammation risk respectively. Comorbidity, in the form of the Charlson Comorbidity Index (CCI), was also examined by one study, which found significantly less comorbidity in female caregivers compared to their male counterparts [47]. One study found no significant sex and gender differences in caregivers’ health related quality of life after controlling for the caregiver’s health, level of depression and amount of medication [56]. Finally, a single study did not find any significant sex and gender differences in self-rated health among caregivers after taking into account various demographic and clinical variables such as caregiver age, education level, BMI, smoking status and health issues [58].

### Qualitative studies

#### Study characteristics

A summary of the four included qualitative studies is presented in Table 2. All of the studies provided information on the sex/gender and age of the participants. All four used semi-structured interviews and were smaller in size, reporting data from a total of 76 caregivers, 42 female and 34 male caregivers respectively. The mean age in studies ranged from 33.6 [53] to 77.6 [54] years and the mean age among all reported samples was 59.5 years. With respect to study setting, two were conducted in a community setting [54, 55], one had a clinical (i.e. tertiary hospital) setting [53] and one was conducted in both clinical and community settings [59]. The type of dementia experienced by the care recipients also varied across the studies. One study included care recipients with Alzheimer’s disease, Parkinson’s dementia or multi-infarct dementia [54]. Another study included care recipients with stage II or III Alzheimer’s disease and related dementia [55]. Finally, one study included care recipients with young-onset frontotemporal lobe dementia [59]. One study did not specify the type of dementia [53].

**Table 2:** Findings of all included qualitative studies.

#	Author Date Country Design Sample by Inclusion criteria (IC) Exclusion criteria (EC)	Population Sample size Age (mean±SD)/ range), yrs Sex/Gender (%M) Dementia Type Assessment criteria Frequencies (%)	Outcome	Analyses Methodology	Results Themes	Notes Limitations
1	Brown et al. 2008 USA Secondary analysis of previous interviews Community IC: >60 years old, caring for spouses with some form of dementia EC: NR	N = 20 Age: 77.6 (range: 63–87) Sex/gender: 45% M AD, PD, multi-infarct dementia NR	Help-seeking patterns	Qualitative content analysis	**Realizing a need for help** Husbands recognize changes and begin seeking help earlier **Facilitating and hindering factors** Both husbands and wives underutilize resources and concerned about being ‘indebted’ to others **Making choices of help-seeking strategies** Husbands were more ‘care managers’ than ‘caregivers’ **Outcomes of help-seeking** Husbands were better able to recognize the importance of having time for themselves Wives described more physical and emotional stress/burden	Limited sample size and demographic (all caregivers were Caucasian and >60 years old)
2	Hayes et al. 2009 USA Intensive interviewing Community IC: spouse diagnosed >6 months prior to interview; demonstrate symptoms of stages II and/or III ADRD EC: NR	N = 28 Age: 67 (M: 74; F: 61) Sex/gender: 46.4% M Stage II and III ADRD NR	Perceptions of identity change and intimacy	Constructivist approach	Husbands continue to view their spouse as wife. Wives begin viewing their husbands as ‘child-like’. *“She’s lost memory skills*, *she’s lost some certain physical skills*, *she can’t focus on things*, *but Kay’s still Kay”* Men expressed more desire for sexual intimacy than women. **Changes in sexual intimacy** In men, attributed to breaking down of spouses’ bodily functions and appearance In women, attributed to changes their spouses’ identity	Possible confounding due to the age difference between men and women Majority Caucasian participants
3	Johannessen et al. 2017 Norway Interviews Clinical and community IC: NR EC: NR	N = 16 Age: 59.6 (Range: 51–69) Sex/gender: 43.8% M Young-onset frontotemporal lobe dementia Psychiatrist or geriatrician diagnosis	Experiences and needs for assistance in daily life	Modified grounded theory	**Shifts in family roles** Men do not seem to be overwhelmed when taking on traditional female roles (e.g. caring, cooking, etc.) while females emphasized the challenges of taking on traditional male roles as the provider and economic organizer of the family	Small sample size Did not consider experiences of the entire family
4	Qadir et al. 2013 Pakistan Semi-structured interviews Clinical IC: NR EC: NR	N = 12 Age: 33.6 (Range: 19–47) Sex/gender: 41.7% M Dementia DSM-IV	Awareness, attitudes and perception of caregiving burden	Thematic analysis	**Physical burden** Women, in particular those that work outside of home, report higher levels of stress compared to men	NR

Abbreviations: AD—Alzheimer’s Diseases; ADRD—Alzheimer’s Disease and Related Dementias; DSM-IV—Diagnostic and Statistical Manual of Mental Disorders, 4^th^ Edition; EC—Exclusion Criteria; IC—Inclusion Criteria; NR—Not Reported; PD—Parkinson’s Disease

#### Caregiving experiences

The four qualitative studies identified sex and gender differences related to caregiving burden, roles, help-seeking patterns as well as perceptions of intimacy among spousal [54, 55, 59] and family (mix of children, grandchildren and spousal) [53] caregivers. A single study identified gender distinctions regarding spousal caregivers’ expressed interest in and feelings about sexual and physical intimacy [55]. In particular, male spousal caregivers continued to pursue sexual relations with their cognitively impaired wife much more frequently than vice versa [55]. Additionally, men expressed more desire for sexual intimacy than women [55]. While males continue to view their spouses as wives, females perceived their husbands as child-like, which led to a decreased interest in emotional and sexual intimacy [55].

Three studies identified higher levels of stress and greater challenges faced by both female spousal and family caregivers [53, 54, 59]. With respect to outcomes of help-seeking, wife caregivers generally described more physical and emotional stress and burden when compared to husband caregivers [54]. In relation to factors contributing to caregiver stress, working female caregivers reported a threefold burden due to their multiple responsibilities in the workplace, at home and as a caregiver [53]. One study highlighted differences in help-seeking behaviors among male and female spousal caregivers within themes relating to the realization of a need to get help, factors that facilitate and hinder help-seeking, making choices of help-seeking strategies and outcomes of help-seeking [54]. Specifically, husbands recognized changes earlier, began help seeking earlier and were better able to recognize the importance of having time for themselves than wives of PWD [54]. Additionally, husbands mostly took on the role of ‘care managers’ and were less likely to provide direct care compared to wives [54]. That said, both groups were found to underutilize the available resources in the family as well as in the community, and were concerned about being ‘indebted’ to others for their help [54]. Finally, a single study explored the shifts in family roles among older spousal caregivers with an average age of 59.6 years. While the authors found male caregivers did not seem to be overwhelmed when taking on traditionally female roles such as caring and cooking, female caregivers emphasized the challenges faced when they had to take on traditional male roles of providing and organizing the family from an economic perspective [59].

## Discussion

This paper systematically reviewed the literature on dementia caregiving between 2007 and 2019 to examine the (1) sex and gender distinctions in caregiving burden experienced by family caregivers of persons with dementia, and (2) sex and gender differences in the impact of caregiving on the physical and mental health of family caregivers of PWD. Among the 13098 articles retrieved in the initial search, only 22 studies were included in the review, which represents a small proportion of the literature in the field. Despite sex and gender being widely collected and reported in studies, few explored the presence and extent of sex and gender differences in caregiving burden. Given that caregivers are mostly female [23], these search results suggest a lack of attention to sex and gender influences in dementia caregiving. However, with more males taking on the caregiving role as women make up the majority of PWD [60, 61], there has been a growing need to understand caregiving experiences from a sex and gender perspective in order to enhance the planning and design of services that would appeal to both male and female caregivers. In the reviewed studies, caregiving burden among family caregivers was measured using various methods with most studies reporting higher burden among females. With respect to mental and physical health, studies examined a wide range of conditions including depression, psychological stress, sense of coherence, ability to control disturbing thoughts, family conflicts, guilt, sleep, quality of life, self-rated health, intimacy experiences, inflammation and comorbidity.

### Sex and gender differences in caregiving burden

Among the 22 studies included in the review, 16 studies examined the sex and gender differences in caregiving burden. This included both formal measures of caregiving burden and semi-structured interviews. Among the 13 studies that used a formal measure to examine the sex and gender differences in caregiving burden, six used the ZBI. Developed more than 30 years ago [62], the ZBI is a reliable and valid caregiving burden instrument most consistently used in dementia caregiving research [63–65]. While all studies that failed to account for confounders found significantly higher levels of caregiving burden among female family caregivers of PWD [39, 45], only half of the studies that did account for confounders (full list shown in Table 1) came to the same conclusion [47, 50]. These contrasting findings highlight the importance of recognizing intersectionality in the context of sex and gender health research. As an analysis approach that moves beyond single or typically favored categories of analysis (e.g. sex, gender, race or class) to consider simultaneous interactions between different aspects of social identity, intersectionality focuses on examining how different socio-demographic factors interact to shape and influence experiences [66, 67]. In the field of caregiving burden, inclusion of these socio-demographic constructs can help to advance understanding of how sex and gender intersects with other dimensions of caregiving. Within this review, a proportion of studies that incorporated additional socio-demographic variables in their analyses and presented an intersectional approach failed to identify significant sex and gender differences, suggesting that these sex and gender distinctions may have been influenced by other socio-demographic factors. As such, these findings call for the adoption of intersectionality plus focus on other social influences in future work on this topic.

Additionally, two studies used the CBI, which like the ZBI, is a scale developed in the late-1980s [68, 69]. The CBI was designed as a diverse, multidimensional and validated instrument to measure the impact of caregiving burden through 24-items selected from a literature review and research [68, 69]. The remaining five studies used the Caregiving Experiences Questionnaire, Pearlin Role Overload Scale, BAS, Pines Burnout Measure and RSS respectively. As most of these scales were developed at a time when caregivers were predominantly female, items within these scales may be inherently gendered and may not adequately reflect the burden and stresses experienced by male caregivers. Moreover, given the myriad of different instruments used by studies to measure caregiving burden, there appears to be a lack of consistency within this topic area, which limits the ability to make comparisons across these studies. As such, future efforts can focus on examining the gendered nature of caregiving burden scales and promoting a level of standardization of the measures used to assess caregiving burden in order to enable meaningful comparisons and knowledge synthesis within this area.

With respect to caregiving burden, findings from the qualitative studies concur with most quantitative studies. Not only did these qualitative studies identify a higher level of stress and challenges faced among female caregivers [53, 54, 59], they also highlighted gendered perspectives that may have contributed to the observed difference between male and female caregivers [54]. Specifically, male caregivers began seeking help earlier and realized the importance of having time to themselves [54]. As such, they were more willing to share some of the caregiving demands and engage in personal activities that provided respite from caregiving. These findings provide a level of insight to the gendered nature of caregiving and its relationship with the observed differences in caregiving burden among male and female caregivers. That said, like most other studies in the review, there was a lack of discussion on the influence of other socio-demographic factors and their role in mediating the relationship between sex, gender and caregiving burden.

Overall, while most of the included studies on caregiving burden demonstrated a higher level of burden among female family caregivers of PWD, these studies often lacked methodological rigor, reflecting the infancy of sex- and gender-based analyses in this area. Specifically, there was a lack of inclusion of other factors that have been shown to influence caregiving burden such as age, time spent on caregiving and dementia severity in the statistical analyses. Given that all but one study are of ‘moderate’ quality, attention should be paid on employing more comprehensive statistical and qualitative methodologies to better tease apart the relationships between sex, gender, as well as other socio-demographic variables and their collective influence on caregiving burden.

### Mental health

Among the five studies that examined the prevalence of depression among family caregivers of PWD, female caregivers reported higher scores on instruments such as the CES-D, BDI and GDS compared to their male counterparts. While these findings are in line with the prevalence of depression in the general population where women are almost twice as likely to be diagnosed with depression than men [70], they may not necessarily reflect variations in the caregiving experiences between males and females. As items within traditional depression scales such as sadness and crying are in conflict with societal ideals of masculinity [71], men may be reluctant to endorse these experiences when completing the depression scales. Additionally, there have been suggestions within the current literature that men’s experiences of depression may manifest with symptoms that are not currently included in traditional depression scales [71]. As such, the scales used in the included studies may not capture the true sex and gender disparities that may be present. Furthermore, the lack of consideration of any confounders in most of the studies’ statistical analyses may also limit the applicability and quality of the evidence. Given the recent development of alternative depression scales that take into account different depressive symptoms among male and females [71], future studies can consider examining the validity and reliability of alternative depression scales for uncovering sex and gender differences in depression within the context of informal caregiving.

The studies in this review also investigated other mental health constructs including psychological distress [42, 56], self-esteem [39], family conflicts [42], self-efficacy [42], guilt [43], psychological quality of life [51] and sense of coherence [56]. In particular, significant sex and gender differences were found in all of the constructs with the exception of self-esteem. Despite having a lack of supporting evidence from other studies within the review, the selection of these mental health constructs among studies suggests their relevance within the caregiving context. Specifically, guilt, self-efficacy and self-esteem have all previously been highlighted as themes arising from caregiving experiences, including but not limited to dementia [72–74]. The paucity of studies that have taken on a sex and gender lens when examining these constructs reflect the current emergence of sex- and gender-based analysis in this area. As such, given the significant differences between male and female caregivers, these findings call for a renewed focus of research to further explore the role of sex and gender in this field.

### Physical health

Studies on sex and gender differences in caregivers’ physical health focused primarily on comorbidities, sleep, inflammation, and intimacy experiences. Significant sex and gender differences were uncovered in most studies with the exception of intimacy experiences, where contrasting findings were found. A single study highlighted the sex and gender difference in comorbidity among family caregivers of PWD. Utilizing the CCI, female caregivers were found to have less comorbidity compared to males. However, there was a lack of any adjustment for confounders such as age, which had been previously identified as an important adjustment when using the CCI [75, 76]. As such, findings from the study ought to be interpreted with caution and more work is needed in this topic area.

With respect to the impact of caregiving on intimacy experiences among spousal caregivers and their care recipients, studies reported contrasting findings. While the quantitative study that examined intimacy using the Experiences of Intimacy with Partner Scale (EOIPS) found no difference between male and female caregivers [41], interviews conducted by Hayes and colleagues uncovered distinctions in the way male and female spousal caregivers view both their partners and sexual intimacy [55]. Given the sensitive nature of the topic, it is expected that caregivers may not be comfortable disclosing their intimate experiences through an open questionnaire. However, they may be more likely to open up about their perspectives on this issue in a safe and private space during an interview. Moreover, with only 3-items, the valid EOIPS merely quantifies the frequency and level of satisfaction of intimate experiences [41]. As such, it may be unable to capture the nuances of these experiences and thoughts that may have driven the different perspectives in male and female caregivers.

One study examined sleep and found significant sex differences in sleep and its related biomarkers including IL-6 and D-dimer. In contrast with other dimensions examined, male caregivers had worse sleep compared to females after accounting for caregiver age, BMI and care recipient’s dementia severity. Sleep plays important functions with respect to physical and psychological restoration, memory and emotional regulation [77]. Poor quality of sleep has been shown to be one of the main problems facing caregivers and tends to significantly impact their caregiving role [78, 79]. Hence, given the lack of literature on the sex and gender differences in caregivers’ sleep experiences, attention should be paid to engage in deeper investigations on the mechanisms underlying these sex and gender differences in sleep and its relationship with caregiving burden in order to develop effective interventions that will better address such an important physical impact of caregiving.

### Limitations

One of the main concerns regarding the included studies was the lack of consistency on the measures used. As such, the estimates provided by each quantitative study were unable to be pooled together and caution is recommended when making inferences. In addition, while socio-demographic variables of the caregivers were extracted, the types of variables are limited to the data collection process of the included studies. As such, certain socio-demographic variables that are known to influence caregiving experiences, such as family income, education level and geographic location may have been omitted by the studies. Moreover, caregiving relationships, which are intricately linked with sex and gender, were not explored in relation to caregiving experiences due to the lack of explicit classifications of the family caregivers in the included studies. Given the limited number of articles retrieved through the systematic search which suggests the overall lack of scholarly focus in this topic area, alternative approaches such as a scoping review may be able to expand the breath of the review to include other caregiving populations.

Given that most of the included studies did not explicitly disentangle sex from gender differences, the review was limited in its ability to report sex and gender findings separately. As such, given the current trend towards disentangling the impacts of sex and gender in understanding male and female differences [80], there is potential for future studies in this field to incorporate a direct gender measure such as the Masculine Gender Role Stress [81] and Bem-Sex-Role-Inventory [82] or construct a gender index based on pre-collected variables. While it is acknowledged that sex and gender interact, these innovative gender measures will enhance our understanding of the relative contribution of sex and gender as individual constructs in differences between male and female caregivers. Finally, this review excluded articles published before 2007, in languages other than English and grey literature. These decisions were made based on the overwhelming number of studies being identified within the databases searched as well as limited empirical evidence about the potential impact of selective searching and inclusion of earlier works on the results of systematic reviews [33]. Despite these limitations, this review aims to enrich science and enhance support provided to family caregivers of PWD by comprehensively pooling together evidence on the sex and gender differences in caregiving burden among family caregivers of PWD.

## Conclusion

To the best of our knowledge, we have conducted a first-of-its-kind systematic review to investigate the sex and gender differences in caregiving burden and its impact on the physical and mental health of family caregivers of PWD. Findings of the included studies suggest the presence of sex and gender differences in caregiving burden, with female caregivers experiencing greater burden compared to their male counterparts. However, given the variety of mental and physical health constructs that were examined by single studies, further research is required to substantiate the evidence. More importantly, the development of a core set of burden scales to be used in studies exploring caregiving burden will enable better comparisons across studies and allow for a more nuanced understanding of the caregiving experience. On a similar note, future work should also take into consideration other socio-demographic and clinical factors such as age, family income, education level, caregiving relationship and dementia severity that may interact with the sex and gender influences in caregiving experiences in order to tease out the nuances in such an intriguing topic area. Specifically, quantitative studies could employ multivariable analyses and qualitative studies could engage in active recruitment of caregivers from a variety of socio-demographic backgrounds. Overall, the current review highlighted a critical gap in the current literature on sex and gender differences in caregiving burden, mental and physical health. While females remain the predominant caregivers at present, there has been a surge in males taking on the role of caregiving in recent years [23, 61]. As such, with the inclusion of more recent articles, this review provides a more contemporary perspective of the distinctions in caregiving experiences between male and females. Nonetheless, more work is needed to enhance our understanding of the nuances in such an intriguing topic area and set the groundwork for future sex- and gender-specific interventions that address the impacts of family caregiving.

## Supporting information

S1 Protocol (PDF)

S1 Checklist (DOC)
