# Supplementary material for: Sex and gender differences in caregiving burden experienced by family caregivers of persons with dementia: A systematic review
Source: PLoS One. 2020 Apr 20;15(4):e0231848. doi: 10.1371/journal.pone.0231848 (PMC7170244; doi:10.1371/journal.pone.0231848)
Supplement: S1 Protocol — (PDF) [file pone.0231848.s001.pdf]

BMJ Open

Sex and gender differences in caregiving burden experienced by family caregivers of persons with dementia: a systematic review protocol

Chen Xiong,[1,2,3] Melissa Biscardi,[1,2,3] Emily Nalder,[1,4] Angela Colantonio[1,4,2,3,5]

To cite: Xiong C, Biscardi M, Nalder E, *et al*. Sex and gender differences in caregiving burden experienced by family caregivers of persons with dementia: a systematic review protocol. *BMJ Open* 2018;**8**:e022779. doi:10.1136/bmjopen-2018-022779

► Prepublication history and additional material for this paper are available online. To view these files, please visit the journal online (http://dx.doi.org/10.1136/bmjopen-2018-022779).

Received 5 March 2018
Revised 9 May 2018
Accepted 6 July 2018

Check for updates

© Author(s) (or their employer(s)) 2018. Re-use permitted under CC BY-NC. No commercial re-use. See rights and permissions. Published by BMJ.

[1]Rehabilitation Sciences Institute, University of Toronto, Toronto, Ontario, Canada
[2]Toronto Rehabilitation Institute, University Health Network, Toronto, Ontario, Canada
[3]Aquired Brain Injury Research Lab, University of Toronto, Toronto, Ontario, Canada
[4]Occupational Science and Occupational Therapy, University of Toronto, Toronto, Ontario, Canada
[5]Dalla Lana School of Public Health, University of Toronto, Toronto, Ontario, Canada

Correspondence to
Chen Xiong;
chen.xiong@mail.utoronto.ca

ABSTRACT

Introduction While much is known about caregiving burden and its consequences on both caregivers and care recipients, reports on the sex and gender differences that may be present among family caregivers of persons with dementia (PWD) are lacking. Attention to and a synthesis of these sex and gender distinctions have direct implications on the planning and development of health services for this population. The current protocol outlines a strategy for a systematic review of the current evidence to identify and synthesise sex and gender distinctions in caregiving burden experienced by family caregivers of PWD.

Methods and analysis A comprehensive search strategy for studies that examine the sex and gender differences in caregiving impacts and experiences has been developed in collaboration with an information specialist at a university. All peer-reviewed English language studies on adult family caregivers of PWD, published from January 2007 to September 2017, found through Medline, Embase, PsycINFO, Cumulative Index to Nursing and Allied Health Literature and bibliographies of identified articles, will be considered eligible. Study quality will be assessed using the Critical Appraisal Skills Programme checklists.

Ethics and dissemination As the first systematic review of its kind that focus on sex and gender differences in caregiving burden, findings will be relevant for healthcare practitioners and researchers who can better prescribe and develop interventions and technologies to better address the specific challenges and burden experienced by male and female family caregivers of PWD. Moreover, given that more than half of family caregivers are females; these sex and gender differences will also be pertinent to policy-makers when evaluating and planning of our healthcare systems to better meet the needs of this population.

Trial registration number CRD42018070032.

Strengths and limitations of this study

- The study of sex and gender differences in family caregiving is important as these distinctions can have significant impacts on the mental and physical health of caregivers.
- To date, there has been no evidence synthesis on the topic of sex and gender differences and caregiving; the protocol outlines a strategy for a study that intends to fill the gap.
- Attention to sex and gender differences in caregiving will set the groundwork and inform research needed to work towards development of tailored interventions for caregivers of different sexes and genders.
- Biases associated with unequal sex and gender distribution among caregivers (ie, majority of family caregivers are female) and exclusion of older articles could not be avoided.
- Systematising data on sex and gender distinctions in caregiving is essential for caregivers, care recipients, healthcare providers, policy-makers and health researchers.

INTRODUCTION

Dementia has become a major public health concern worldwide, with an annual total economic burden of more than US$818 billion.[1] Approximately, 47 million individuals are living with dementia-related conditions, and by 2050, that number is expected to increase to more than 131 million as the population ages.[1] Dementia is an umbrella term that refers to a range of conditions all characterised by a decline in mental ability, the most common being Alzheimer's disease.[2] In addition to symptoms associated with cognitive decline, persons with dementia (PWD) also experience behavioural and psychological disturbances such as depressive mood, anxiety, restlessness, agitation among others.[3 4]

The rising prevalence of dementia will also mean that a growing number of families are providing in-home care for older adults with dementia.[5] While the presence of kinship and the familiarity of the homecare environment has been shown to facilitate the caregiving process and delay unfavourable health outcomes such as death or institutionalisation, caregiving for PWD remains a highly stressful experience for these family caregivers and may contribute to negative mental health and an increased risk of mortality and

serious illness.[6 7] Defined as the strain or load borne by a person who cares for a chronically ill, disabled or elderly family member, caregiving burden is a multidimensional response to the physical, psychological, emotional, social and financial stressors associated with the caregiving experience.[8 9] As a result, it can have devastating and long-term effects on the physical, social, emotional and financial status of family caregivers of PWD.[10 11] Previous research on caregiver burden had shown an association with psychological symptoms such as depression in both the caregiving population.[12 13] Similarly, physical conditions such as hyperlipidaemia, hyperglycaemia and insufficiency of the cellular immune system may also develop as a consequence caregiving burden.[14 15]

Given its multidimensional nature, factors contributing to caregiver burden are diverse and can vary significantly between different caregivers. In particular, older age, lower socioeconomic status and poor access to/low acceptance of social support among caregivers had all been associated with greater caregiver burden.[12] Additionally, adult children of PWDs were found to be less affected burdened by caregiving when compared with their spousal counterparts, highlighting the effect of family relationships on experiences of caregiving burden.[16]

Unfortunately, while much is known about caregiving burden and its consequences on both the caregivers and care recipients, there is a paucity of information on the sex and gender differences that may be present between male and female family caregivers. Within the context of this systematic review, sex represents a set of biological attributes in humans associated with physical and physiological features, while gender constitutes the socially constructed roles, behaviours, expressions and identities of girls, women, boys, men and gender diverse individuals.[17] While these constructs are distinct, we recognise that they are on a continuum and inter-related. As such, both constructs will be referred as 'sex and gender' for the remainder of this paper.

The limited literature on sex and gender influences on caregiving burden has found differences in well-being, psychosocial and overall health status between male and female caregivers through cross-sectional surveys.[18] Additionally, differences were also observed in relation to subjective measures of health and well-being, with female caregivers reporting a greater perception of ill health and lower levels of quality of life than male caregivers.[19] Similarly, the forms of caregiver burden and stress experienced were also heavily influenced by sex distinctions. While male caregivers were found to experience a low morale and a greater need for social support, the burden experienced by female caregivers was often attributed to their relationships with other family members.[9]

Despite the fact that several studies have attempted to incorporate a sex and gender perspective in the field of caregiving burden, there has been no study conducted to identify and collect all of the sex and gender differences in caregiving burden highlighted within the existing literature. Given that more than half of family caregivers are female; these differences are paramount for evaluating and planning of our healthcare systems to better meet the needs of this population.[20] This also has implications for healthcare practitioners and researchers, who would be better able to accurately and appropriately develop interventions and technologies to better address the specific challenges and burden experienced by male and female family caregivers of PWD. As such, this protocol is for a systematic review of the literature with the main objective to describe the sex and gender distinctions in caregiving burden experienced by family caregivers of PWD. Specifically, the secondary objective of the systematic review is to determine the impact of sex and gender differences in caregiving burden on the physical and mental well-being among family caregivers of PWD.

METHODS

The systematic review that this protocol describes will be conducted and reported in compliance with the Preferred Reporting Items for Systematic Reviews and Meta-Analyses (PRISMA) guidelines.[21] In accordance with these guidelines, this systematic review protocol was registered with the International Prospective Register of Systematic Reviews on 8 February 2018. The Sex and Gender in Systematic Reviews Planning Tool was also used during the protocol development process to better facilitate the integration of sex and gender concepts.[22]

Search strategy

The following databases will be searched for relevant articles:

1. MEDLINE and MEDLINE In-Process via Ovid.
2. Embase via Ovid.
3. PsycINFO via Ovid.
4. Cumulative Index to Nursing and Allied Health Literature via EBSCO.

The search strategy was developed through consultations with librarians and experts in the field of caregiving and dementia. The search strategy will use both text and index terms capture each of the three concepts, 'family caregiver', 'dementia' and 'burden'. Due to the lack of conceptual clarity on caregiving burden, the search will also include similar concepts such as 'strain' and 'stress' among others. Boolean Operators AND and OR will be used to combine search concepts and search terms within each concept, respectively. A full version of the search strategy associated with each of the above databases is provided in the supplemental document (online supplemental table S1).

Recognising that most informal caregivers are family and potential distinctions in the caregiving experiences of familial and non-familial caregivers, this review focuses specifically on family caregivers. While most family caregivers for PWD are spouses, partners and adult children, this systematic review will include all members of the nuclear and extended family including but not limited to grandchildren, siblings, cousins, nieces, nephews. A

Xiong C, et al. BMJ Open 2018;8:e022779. doi:10.1136/bmjopen-2018-022779

complete list of the type of family members included is reflected in the search strategy. Given that dementia is an overall term that describes a wide range of symptoms associated with a decline in mental ability, the search strategy has been expanded to include all types of dementia including Alzheimer's disease, which accounts for the majority of dementia cases.

Inclusion and exclusion criteria

A first screen, the title and abstract screen, will be conducted by two independent reviewers on all retrieved articles. Articles that pass the first screen must be written in the English language, peer reviewed, published between January 2007 and September 2017, meet all of the following inclusion criteria and have a full-text version available for review:

1. Examine the experiences and/or impacts of caregiving among family caregivers of individuals with any form of dementia.
2. Report sex and/or gender distribution of study population and/or report results stratified by sex and/or gender.
3. Include both male and female family caregivers of PWD.

On the other hand, articles will be excluded if they are case reports, abstracts, conference material and editorials or commentaries as these sources are unlikely to present the information in the level of detail required for the purposes of this systematic review. Theses will also be excluded as they are not peer reviewed. In addition, articles that include both family and formal caregivers but do not stratify findings by caregiver type or do not report results specifically for care recipients with some form of dementia will also be excluded. Finally, articles that examine the effect of various interventions on caregiving burden will also be excluded from the review. Articles that meet the above inclusion and exclusion criteria will be included for a second screen, which will be a full-text screen. During the second screen, assessment of the full text by two independent reviewers will determine if studies will be included in the review. Articles that are included in the systematic review must examine caregiving burden experienced specifically by family caregivers of PWD. In addition, included articles must also stratify and report on the findings by sex and/or gender. At both screening stages, two independent reviewers will assess eligibility and differences in opinion will be resolved by group discussions with a third reviewer with the goal of reaching consensus.

The reference lists of included full-text articles will also be handsearched. Experts in the field of family caregivers and caregiving burden will be consulted to ensure that no additional studies are missed with the use of the above search strategy and inclusion/exclusion criteria. The study selection process along with reasons for exclusions at the full-text level will be presented using a PRISMA study flow diagram. In addition, the number and type (qualitative or quantitative) of studies that addressed each of the review objectives will also be reflected within the PRISMA diagram.

Data extraction and quality assessment

Study data will be abstracted by two reviewers. The standardised data extraction table will include author and publication year, study purpose, geographical location of study, setting, demographics of the study caregiver population including but are not limited to, age, caregiving relationships, race/ethnicity and income level, sample size, type of dementia, sex and gender breakdown of study participants, study design, study methodology, results related to caregiving experiences (caregiving burden and impacts on physical and/or mental health) as well as sex and gender differences in those experiences. To address the objectives of this review, sex and gender differences in caregiver burden among family caregivers of PWD will be listed in the column titled 'sex and gender differences'. This information will provide a starting point to identify findings from included studies that will be used to address the review's specific objectives.

Quality assessment of the studies will be conducted by two reviewers independently using the Critical Appraisal Skills Programme (CASP) checklists.[23] The checklists can be applied to various study designs including systematic reviews, randomized controlled trials, cohort and case–control studies, which will allow for the assessment of the range of study designs that may be included in this systematic review. Specifically, the CASP Qualitative checklist will be used for all qualitative studies included in the review. Cohort and case–control studies will be assessed using the CASP Cohort and case–control checklists, respectively. Methodological quality, such as the use of appropriate statistical tests, accounting for confounding and the sample or population selection, is of particular importance as it can affect the interpretations and generalisability of results. Disagreements between the review authors over the quality assessment of particular studies will be resolved by discussion, with the involvement of a third review author where necessary.

Analyses

Analyses of the included articles will be determined by the search results and included studies. At the first stage of analysis, included studies that address each of the review objectives will be grouped together. Due to the paucity of information in this developing field, a narrative synthesis guided by the Guidance for Narrative Synthesis in Systematic Reviews will be conducted.[24] In particular, the techniques of 'textual descriptions', 'tabulation' and 'grouping and clusters' will be used within the analyses. As a common approach in all types of systematic reviews, tabulation involves presenting study data visually in tabular form and is useful in developing an initial description of the included studies as well as identifying patterns and themes across studies. Within each group of studies, data extracted from the included articles will be tabulated as per the categories defined in the data extraction section. To further aid the

process of description and analysis, extracted data will be grouped into clusters based on variables such as research type (eg, qualitative or quantitative, with mixed-methods studies classified based on their primary methodology and outcome measurement), study design (eg, case–control, cohort, focus groups, qualitative, etc), type of dementia, as well as outcome (eg, physical and/or mental health). Synthesis of the extracted data will involve summarisation and explanation of the sex and gender differences for the included studies based on the grouping variables. Finally, the quality of the included studies will also be described as part of the narrative synthesis.

In addition, quantitative studies that address each of the review objectives will, when possible, be pooled in statistical meta-analysis separately. Specifically, quantitative data including ORs, relative risks, HRs and weighted mean differences with their 95% CIs will be extracted and analysed. Since the investigation of sex and gender differences in caregiving burden among family caregivers is still an emerging field, there has yet to be an established gold-standard measurement for sex and gender differences in the prevalence, impact and forms of caregiving burden within this population. As such, it is expected that a variety of outcome measures will be used across studies to identify sex and gender differences in caregiving burden, In addition, other aspects of a study such as the clinical and demographic characteristics of participants, geographical location and type of statistical analyses used are also expected to vary across each selected article. Together, these variations contribute to heterogeneity across the included studies, which is one of the factors that determine the appropriateness of conducting a meta-analysis. As such, random-effects models will be used to confirm the presence of heterogeneity across studies that address each review objective. Heterogeneity will be assessed using the I^2 statistic. P values of 0.05 or less will be considered as statistically significant and a meta-analysis will only be performed when appropriate.

Finally, synthesised findings of qualitative and quantitative findings addressing each review objective will be aggregated, as appropriate. This will be done through a narrative comparison of the quantitative and qualitative findings. Specifically, themes and concepts arising from both quantitative and qualitative synthesis will be assembled and examined to produce a set of synthesised findings associated with sex and gender distinctions in caregiving for PWD.

Patient and public involvement

Patients and the public were not involved in this project.

DISCUSSION

Implications

This systematic review aims to investigate the sex and gender distinctions in caregiving burden experienced by family caregivers of PWD, specifically the prevalence, forms and impacts of caregiving burden on family caregivers. As the first systematic review of its kind that focus on sex and gender differences in caregiving burden, findings from this review will be relevant for healthcare practitioners and researchers as who can better prescribe and develop interventions and technologies to better address the specific challenges and burden experienced by male and female family caregivers of PWD. Moreover, given that more than half of family caregivers are females; these sex and gender differences will also be pertinent to policy-makers when evaluating and planning of our healthcare systems to better meet the needs of this population. On completion of the review, a manuscript will be submitted to a peer-reviewed journal to be identified at a later stage for publication. In addition, findings from the review will also be presented at various national and international conferences to better ensure that the results from the review reach their intended knowledge users.

Strengths and limitations

A major strength of this study is the integration of both qualitative and quantitative data, which allows for a more comprehensive synthesis of available evidence on sex and gender differences in family caregiving for PWD. In addition, risk of bias assessments for individual study types will allow for the detection of major flaws in each included study and these inform future research on caregiving burden among family caregivers of PWD. On the other hand, one limitation of the review relate to the inclusion and exclusion criteria of the title and abstract screen. Including only abstracts that provide sex and/or gender distribution of study participants and/or report results stratified by sex and/or gender may overlook a small number studies that only provided the information in the full-text article. In addition, while sociodemographic variables of the caregivers will be extracted, the types of variables are limited to the data collection process of the included studies. As such, certain sociodemographic variables that are known to influence caregiving experiences may be omitted. Finally, this study excluded articles published before 2007, in languages other than English and grey literature. These decisions were made based on the overwhelming number of studies being identified within the databases searched as well as limited empirical evidence about the potential impact of selective searching and inclusion of earlier works on the results of systematic reviews.[25] Despite these limitations, this protocol is for a review that comprehensively synthesises evidence on the sex and gender differences in caregiving burden among family caregivers of PWD, aiming to enrich science and enhance support provided to family caregivers of PWD.

Acknowledgements We gratefully acknowledge the involvement of Erica Lenton, Faculty Liaison and Instruction Librarian at the University of Toronto for her assistance in development of the comprehensive literature search.

Contributors Protocol concept and design: CX and AC. Registry PROSPERO: CX. Acquisition of data: CX. Administrative, technical and material support: CX, MB and

EN. Statistical analysis approach: CX and AC. Drafting of the manuscript: CX. Critical revision of the manuscript for important intellectual content: All authors.

Funding The study was funded by the Canadian Institutes of Health Research (CIHR) under Grant SGB—155385 (PI: AC). AC is supported by the Canadian Institutes for Health Research Grant–Institute for Gender and Health (#CGW-126580). CX is supported by the Peterborough K.M. Hunter Charitable Foundation Graduate Award.

Disclaimer The funders had no role in study design, data collection, decision to publish or preparation of the manuscript.

Competing interests None declared.

Patient consent Not required.

Provenance and peer review Not commissioned; externally peer reviewed.

Open access This is an open access article distributed in accordance with the Creative Commons Attribution Non Commercial (CC BY-NC 4.0) license, which permits others to distribute, remix, adapt, build upon this work non-commercially, and license their derivative works on different terms, provided the original work is properly cited, appropriate credit is given, any changes made indicated, and the use is non-commercial. See: http://creativecommons.org/licenses/by-nc/4.0/.

REFERENCES

1. Prince M, Comas-Herrera A, Knapp M, *et al*. *World Alzheimer Report 2016*. London, United Kingdom, 2016.
2. Alzheimer's Association. What is Dementia? 2018 https://www.alz.org/what-is-dementia.asp (accessed 22 Jan 2018).
3. Clinic M. Dementia. Symptoms and Causes. 2017 https://www.mayoclinic.org/diseases-conditions/dementia/symptoms-causes/syc-20352013 (accessed 22 Jan 2018).
4. World Health Organization. Dementia. 2015 http://www.who.int/mediacentre/factsheets/fs362/en/ (accessed 22 Jan 2018).
5. Alzheimer's Society of Canada. *Rising Tide: The Impact of Dementia on Canadian Society*. Toronto, Ontario, Canada, 2010.
6. Givens JL, Mezzacappa C, Heeren T, *et al*. Depressive symptoms among dementia caregivers: role of mediating factors. *Am J Geriatr Psychiatry* 2014;22:481–8.
7. Hughes TB, Black BS, Albert M, *et al*. Correlates of objective and subjective measures of caregiver burden among dementia caregivers: influence of unmet patient and caregiver dementia-related care needs. *Int Psychogeriatr* 2014;26:1875–83.
8. Stucki BR, Mulvey J. *Can Aging Baby Boomers Avoid the Nursing Home: Long-term Care Insurance for" aging in Place"*: American Council of Life Insurers, 2000.
9. Etters L, Goodall D, Harrison BE. Caregiver burden among dementia patient caregivers: a review of the literature. *J Am Acad Nurse Pract* 2008;20:423–8.
10. Ankri J, Andrieu S, Beaufils B, *et al*. Beyond the global score of the Zarit Burden Interview: useful dimensions for clinicians. *Int J Geriatr Psychiatry* 2005;20:254–60.
11. Vellone E, Piras G, Talucci C, *et al*. Quality of life for caregivers of people with Alzheimer's disease. *J Adv Nurs* 2008;61:222–31.
12. Pinquart M, Sörensen S. Correlates of physical health of informal caregivers: a meta-analysis. *J Gerontol B Psychol Sci Soc Sci* 2007;62:P126–P137.
13. Varela G, Varona L, Anderson K, *et al*. Alzheimer's care at home: a focus on caregivers strain. *Prof Inferm* 2011;64:113.
14. Vitaliano PP, Russo J, Niaura R. Plasma lipids and their relationships with psychosocial factors in older adults. *J Gerontol B Psychol Sci Soc Sci* 1995;50:P18–P24.
15. Mills PJ, Adler KA, Dimsdale JE, *et al*. Vulnerable caregivers of Alzheimer disease patients have a deficit in beta 2-adrenergic receptor sensitivity and density. *Am J Geriatr Psychiatry* 2004;12:281–6.
16. O'Rourke N, Kupferschmidt AL, Claxton A, *et al*. Psychological resilience predicts depressive symptoms among spouses of persons with Alzheimer disease over time. *Aging Ment Health* 2010;14:984–93.
17. Canadian Institutes of Health Research. Definitions of Sex and Gender. 2015 http://www.cihr-irsc.gc.ca/e/47830.html (accessed 4 Sep 2017).
18. Chiou CJ, Chen IP, Wang HH. The health status of family caregivers in Taiwan: an analysis of gender differences. *Int J Geriatr Psychiatry* 2005;20:821–6.
19. Yee JL, Schulz R. Gender differences in psychiatric morbidity among family caregivers: a review and analysis. *Gerontologist* 2000;40:147–64.
20. Cranswick K, Dosman D. Eldercare: what we know today. *Canadian social trends* 2008;86:49–57.
21. Moher D, Liberati A, Tetzlaff J, *et al*. Preferred reporting items for systematic reviews and meta-analyses: the PRISMA statement. *PLoS Med* 2009;6:e1000097.
22. Doull M, Welch V, Puil L, *et al*. Development and evaluation of 'briefing notes' as a novel knowledge translation tool to aid the implementation of sex/gender analysis in systematic reviews: a pilot study. *PLoS One* 2014;9:e110786.
23. Critical Appraisal Skills Programme. CASP Checklists. 2017 http://www.casp-uk.net/#!casp-tools-checklists/c18f8 (accessed 22 Jan 2018).
24. Popay J, Roberts H, Sowden A, *et al*. Guidance on the conduct of narrative synthesis in systematic reviews. *A product from the ESRC methods programme Version* 2006;1:b92.
25. Hartling L, Featherstone R, Nuspl M, *et al*. Grey literature in systematic reviews: a cross-sectional study of the contribution of non-English reports, unpublished studies and dissertations to the results of meta-analyses in child-relevant reviews. *BMC Med Res Methodol* 2017;17:64.
